# Missing observations in regression: a conditional approach

**DOI:** 10.1098/rsos.220267

**Published:** 2023-02-08

**Authors:** H. S. Battey, D. R. Cox

**Affiliations:** ^1^ Department of Mathematics, Imperial College London, London SW7 2AZ, UK; ^2^ Nuffield College, University of Oxford, Oxford OX1 1NF, UK

**Keywords:** ancillarity, EM algorithm, fractional factorial, Hadamard matrix, missing data, regression

## Abstract

This note presents an alternative to multiple imputation and other approaches to regression analysis in the presence of missing covariate data. Our recommendation, based on factorial and fractional factorial arrangements, is more faithful to ancillarity considerations of regression analysis and involves assessing the sensitivity of inference on each regression parameter to missingness in each of the explanatory variables. The ideas are illustrated on a medical example concerned with the success of hematopoietic stem cell transplantation in children, and on a sociological example concerned with socio-economic inequalities in educational attainment.

## 1. Introduction

Analysis of observational data is frequently hindered by observations on some explanatory variables being missing. There is a rich literature on this, stemming indirectly from the self-consistency property of maximum likelihood estimation [1], which is perhaps best approached via Efron [2,3, p. 351]. This, in particular, forms the basis for the EM algorithm [4,5] for maximum likelihood estimation from incomplete data. In a regression context when the missing observations are on covariates, application of these ideas necessitates specification of a joint probability model for the vector of responses *Y* and the matrix of covariates *X*, which leads essentially to a probability model for the missing observations. Efficient algorithms are in fact rather similar to imputation methods, based on an assumption that observations are missing at random, not to be confused with missing completely at random, an even stronger assumption.

Modelling aspects of the distribution of *X*, or indeed the joint distribution of *X* and *Y*, is in contradiction to the practice of conditioning on *X* in a regression setting. At least when data are fully observed, the latter is appropriate based on ancillarity considerations, since the regression function is a property of the conditional distribution of *Y* given *X*, so that the distribution of *X* is irrelevant. In fact, when the *d*-dimensional vector of regression coefficients is a canonical parameter of an exponential family, the appropriate conditional calculation for the parameter of interest, the *d*th coefficient say, conditions on the realized values of $\left(x_{\cdot 1}^{T}Y,\ldots ,x_{\cdot (d-1)}^{T}Y\right)$, where *x*_·*j*_ denotes the *j*th column of *X* and $x_{\cdot j}^{T}$ is its transpose. This choice both eliminates the nuisance parameters and ensures that the precision attached to the conclusions is that actually achieved and not an average over hypothetical, the so-called recognizably distinct, situations that have not in fact occurred. A detailed discussion of these issues is not necessary for present purposes. See e.g. [6, §IV.4] or [7, §4.2].

This short article illustrates what we consider to be a more appropriate approach to presenting conclusions when covariate data are missing. This entails replacing unobserved entries by rather extreme assignments in such a way that the effect of missingness of each covariate can be assessed. One of several conclusions emerges: that the missing observations do not materially affect inference on the aspects of interest; that the missingness in some variables affects inference on some but not all of the regression coefficients; or that the missingness is so severe that reliable conclusions are not possible without strong uncheckable assumptions on the process through which the missingness arises. The focus is primarily on the main effects of missingness, estimated from a simple fractional factorial design for the missing entries of *X*, although in principle interactive effects can also be assessed, provided that the number of columns of *X* with missing entries is not impractically large. The idea appears partially, in less explicit form, in the example application of Battey *et al.* [8].

## 2. Full and fractional factorial assessments of missingness

Let *x*_*i*_ for *i* = 1, …, *n* be *d*-dimensional vectors, the transposed rows of an *n* × *d* covariate matrix *X* and let *y*_*i*_ be the associated outcome variables. For a subset $\overset{\circ }{I}\subset \{1,\ldots ,n\}$ of observations, some entries of *x*_*i*_ are missing for reasons that are unknown. The set of observed vectors are denoted by $\overset{\circ }{\underset{i}{x}}$, for *i* = 1, …, *n*. Note that $\overset{\circ }{\underset{i}{x}}=x_{i}$ for $i\notin \overset{\circ }{I},$ and otherwise $\overset{\circ }{\underset{i}{x}}$ has at least one entry missing.

Suppose that the missing entries arise in *m* ≤ *d* of the columns of $\overset{\circ }{X}$, where $\overset{\circ }{X}$ is the *n* × *d* matrix with ${\overset{\circ }{x}}_{i}^{T}$ as its rows. Consider initially replacing all missing entries from a single column of $\overset{\circ }{X}$ by the maximum or minimum of the observed entries in that column. This can in principle be done for all 2^*m*^ combinations of high and low values, leading to 2^*m*^ matrices, ${\overset{\circ }{X}}^{\left(c\right)}$ say, whose transposed rows are denoted by ${\overset{\circ }{x}}_{i}^{\left(c\right)},$ where *c* = 1, …, 2^*m*^ indexes a particular column-wise combination of substitutions for the missing entries of $\overset{\circ }{X}$. Associated with these are 2^*m*^ vectors of regression coefficient estimates, written ${\hat{\beta }}^{\left(1\right)},\ldots ,{\hat{\beta }}^{\left(2^{m}\right)}$, each in $R^{d}$, and their estimated standard errors. For each entry of the unknown regression coefficient vector, the main effect of missingness of each variable and any low-order interactive effects can be estimated by the appropriate factorial contrast.

It is not critical that the maximum and minimum values of each observed column be used in the combinatorial replacements, only that relatively extreme assignments be made to assess the sensitivity, where ‘extreme’ is calibrated against the data that have been observed. In the study by Battey *et al.* [8], the upper and lower quartiles of any continuous variables were used, giving a less conservative assessment. A caveat concerns the situation in which extremity is the reason for the missingness, for instance, if a measuring device is unable to record observations above or below a certain threshold. In that case, the analysis would not faithfully reflect the range of conclusions that could have been reached had the data been available in their entirety. Since the reason for the missingness is known, it would be more sensible in that case to use pairs of values at and exceeding the threshold in the unobservable direction.

As elucidated in appendix A for linear least squares, it cannot be guaranteed in general that the inaccessible estimate $\hat{\beta }$, based on the unobservable matrix *X*, is contained in the convex hull of ${\hat{\beta }}^{\left(1\right)},\ldots ,{\hat{\beta }}^{\left(2^{m}\right)}$. The sensitivity analysis to be outlined is a way of presenting the evidence in an incisive form, while being transparent about the limitations of the data. By contrast, imputation procedures seek to present inferential statements as if the missing data had been observed. McCullagh [9, Chapter 14] convincingly articulates the matter and concludes that the practice can be rather misleading, a view that we share. In the present context, it is also, at least from one point of view, a violation of conditionality as noted earlier.

### 2.1. Illustration for two columns with missing entries

Suppose for illustrative simplicity that there are two columns with missing entries among those of the *n* × *d* matrix *X*. It is helpful to introduce in this illustration a more explicit notation coinciding with the usual one for factorial designs. Thus, we associate with the incomplete columns of *X* the factors *A*, *B* and a general treatment combination *a*^*j*^ *b*^*k*^, where *j* and *k* take values zero and one when the corresponding factor is at its high and low level, respectively. Thus, when *j* = *k* = 1, the missing entries in both columns are replaced by the maximum of the column entries that have been observed. The treatment combination of all factors at their low level is written (1). The four treatment combinations are *X*^(1)^, *X*^(*a*)^, *X*^(*b*)^ and *X*^(*ab*)^ and what would be called outcome variables in the usual experimental design terminology are the *d*-dimensional vectors ${\hat{\beta }}^{\left(1\right)},{\hat{\beta }}^{\left(a\right)},{\hat{\beta }}^{\left(b\right)}$ and ${\hat{\beta }}^{\left(ab\right)}$ and their estimated standard errors. The effect of missingness in the column associated with *A* is then summarized by the usual factorial contrast for the main effect of *A*, namely, $2{\hat{\tau }}^{A}$, where

2.1 $${\hat{\tau }}^{A}=\frac{\left({\hat{\beta }}^{\left(ab\right)}+{\hat{\beta }}^{\left(a\right)}-{\hat{\beta }}^{\left(b\right)}-{\hat{\beta }}^{\left(1\right)}\right)}{4}$$

and similarly,

$${\hat{\tau }}^{B}=\frac{\left({\hat{\beta }}^{\left(ab\right)}+{\hat{\beta }}^{\left(b\right)}-{\hat{\beta }}^{\left(a\right)}-{\hat{\beta }}^{\left(1\right)}\right)}{4}$$

and

$${\hat{\tau }}^{AB}=\frac{\left({\hat{\beta }}^{\left(ab\right)}-{\hat{\beta }}^{\left(b\right)}-({\hat{\beta }}^{\left(a\right)}-{\hat{\beta }}^{\left(1\right)})\right)}{4}.$$

These are not to be interpreted as estimators of any population-level quantities as they are considered conditionally on the data. The factorial contrast $2{\hat{\tau }}^{A}$ is the effect of changing *A* from its low to its high level, averaged over the levels of *B*, and $2{\hat{\tau }}^{AB}$ is the difference between the effects of changing *A* when *B* is at its high level and when it is at its low level. Effectively, the contrast for variable *j* is a discrete approximation to the partial derivative of $\hat{\beta }$ with respect to the aspects of *x*_·*j*_ that have not been observed, holding all other such aspects fixed.

### 2.2. Generalization to higher-dimensional missingness

If *m* is small, it is reasonable to report the (m+(m2))×d-dimensional matrix of main effects of missingness and pairwise interactions. When the number *m* of covariates subject to missing observations exceeds 4 or 5, the 2^*m*^ sets of coefficients from the full factorial is unreasonably large for presentation, and wasteful for constructing the *m* main effects of missingness. These may instead be obtained from a 2^*m*−ℓ^ fractional factorial arrangement with ℓ such that 2^*m*−ℓ^ ≥ *m* + 1. For a comprehensive discussion of fractional factorial arrangements, conveniently studied using prime power commutative groups of order 2^*m*^, see Cox and Reid [10, §5.5.2] or Bailey [11, §13]. When interest lies in main effects only, construction is greatly simplified by using Hadamard matrices, with a Hadamard matrix of dimension 2^*m*^ being constructed from one of half the size as follows:

2.2 $$H_{2^{m}}=\left(\begin{array}{cc} H_{2^{m-1}} & \;H_{2^{m-1}}\\ H_{2^{m-1}} & -H_{2^{m-1}} \end{array}\right).$$

The 2^*m*^ rows of the sub-matrix

$$\left(\begin{array}{c} \;H_{2^{m-1}}\\ -H_{2^{m-1}} \end{array}\right),$$

after discarding the first column, define the treatment combinations associated with the full factorial, and the 2^*m*−1^ rows of the sub-matrices $H_{2^{\left(m-1\right)}}$ and $-H_{2^{\left(m-1\right)}}$, after discarding the first column of each, define the treatment combinations associated with the two distinct half-replicates of the full factorial system. For instance, with *m* = 3,

H2m=( 1  1  1  1  1  1  1  1 1−1  1−1  1−1  1−1 1  1−1−1  1  1−1−1 1−1−1  1  1−1−1  1 1  1  1  1−1−1−1−1 1−1  1−1−1  1−1  1 1  1−1−1−1−1  1  1 1−1−1  1−1  1  1−1),

and the assignments associated with the two half-replicates are {*X*^(*abc*)^, *X*^(*b*)^, *X*^(*a*)^, *X*^(*c*)^} and {*X*^(1)^, *X*^(*ac*)^, *X*^(*bc*)^, *X*^(*ab*)^}. Orthogonality of the Hadamard sub-matrices ensures that each factor is present twice at each level with a different combination of levels for the other factors.

More generally, for ℓ such that 2^*m*−ℓ^ ≥ *m* + 1, there are ℓ fractions of size 2^−ℓ^ of the full 2^*m*^ factorial design, and two distinct Hadamard matrices of dimension 2^*m*−ℓ^ embedded within a given one of dimension 2^*m*^. The last *m* columns specify (2^−ℓ^)-replicates of the full factorial experiment. For example, with *m* = 5, a full factorial entails 32 treatments. The highest available degree of fractionation gives two quarter-replicates specified by the eight rows and last five columns of the two distinct Hadamard sub-matrices of *H*_16_ (or −*H*_16_). In view of this, when *m* is large, the main effects of missingness can be constructed analogously to equation (2.1) by assigning high and low entries as determined by the last *m* columns of a Hadamard matrix of dimension *M*, say, where *M* ≥ *m* + 1.

Let $F\subset \{1,\ldots ,2^{m}\}$ denote a treatment combination associated with, say, a half replicate of the full factorial, as detailed earlier. The main effects of missingness could be constructed either from F or its complement $\bar{F}=\{1,\ldots ,2^{m}\}\setminus F$. The answers will be similar provided that strong interactive effects are not present. Since appreciable discrepancies can be checked for, fractional replication provides an internal mechanism for validating or refuting the reasonableness of the simplified analysis.

Two further comments are helpful. If the Hadamard matrix is not in the standard form (2.2), the column consisting only of 1 or −1 should be discarded. If *M* > *m* + 1, any of the redundant columns can also be used to estimate main effects, and the conclusions will be consistent, as these specify the same treatment combinations in different orders.

There results an *m* × *d* matrix of main effects of missingness, where the (*j*, *k*)th entry is the effect of missing observations on variable *j* on the *k*th estimated regression coefficient. Whether a particular main effect of missingness is deemed large should be calibrated against the estimates themselves so as to make the assessment dimension free. This can be done for both the coefficient estimates and the estimates of standard errors. The ideas are best illustrated with an example; see §3.

## 3. Two examples

### 3.1. A medical example

The ideas are illustrated using publicly available data from Kalwak *et al.* [12] on the success of hematopoietic stem cell transplantation in children. The measured covariates in this study included treatment modifications and numerous forms of mismatch between donors and recipients. This was a wide-ranging investigation, and we focus on one aspect of it. In particular, the binary outcome, coded as {0, 1} = {unsuccessful, successful}, classifies the treatment as unsuccessful if the patient died or relapsed within the follow-up period. The shortest such period for a surviving patient was 433 days. The covariate data are summarized in table 1, where *x*_·*j*_ represents the *j*th column of the covariate matrix *X*. Key treatment variables are *x*_·1_ to *x*_·4_, which measure whether the stem cells were sourced from bone marrow or peripheral blood and the relative and absolute concentrations of CD3^+^ and CD34^+^ cell doses after infusion. Variables *x*_·5_ to *x*_·8_ measure the degree of compatibility between donor and recipient, with *x*_·5_ and *x*_·6_ specifying the number of antigens and alleles in which donor and recipient differ, *x*_·7_ indicating whether their blood groups match, and *x*_·5_ a measure of serological compatibility according to cytomegalovirus infection before transplantation (the higher the value, the lower the compatibility). Variables *x*_·9_ to *x*_·18_ are intrinsic features of the donor and recipient whose interpretations are mostly clear from table 1. Exceptions are *x*_·9_ and *x*_·10_, indicating the presence or absence of cytomegalovirus infection in, respectively, the recipient and donor of hematopoietic stem cells before transplantation, and *x*_·11_, indicating the presence of the Rh factor on the recipient’s red blood cells.

**Table 1.** Summary of data.

covariate	description	sample range	% missing
*x*_·1_	stem cell source	{0 = marrow, 1 = peripheral blood}	0
*x*_·2_	CD3^+^/CD34^+^	[0.20−99.56]	2.67
*x*_·3_	CD3^+^ per kg	[0.040−20.02]	2.67
*x*_·4_	CD34^+^ per kg	[0.79−57.78]	0
*x*_·5_	antigen discrepancies	{0, 1, 2, 3}	0.53
*x*_·6_	allele discrepancies	{0, 1, 2, 3, 4}	0.53
*x*_·7_	blood group match	{0 = mismatch, 1 = match}	0.53
*x*_·8_	CMV incompatibility score	{0, 1, 2, 3}	8.56
*x*_·9_	recipient CMV	{0 = absent, 1 = present}	7.49
*x*_·10_	donor CMV	{0 = absent, 1 = present}	1.07
*x*_·11_	recipient Rh factor	{0 = absent, 1 = present}	1.07
*x*_·12_	recipient body mass	[6, 103]	1.07
*x*_·13_	previous relapse	{0 = no, 1 = yes}	0
*x*_·14_	risk group	{0 = standard, 1 = high}	0
*x*_·15_	disease type	{0 = non-malignant, 1 = malignant}	0
*x*_·16_	recipient sex	{0 = female, 1 = male}	0
*x*_·17_	recipient age (years)	[0.60, 20.2]	0
*x*_·18_	donor age (years)	[18.65, 55.55]	0

Although these data appear to have been carefully collected, a small number of entries for 10 of the 18 variables are missing for reasons that are unknown. To quantify the effects of missingness on the estimates of the logistic regression coefficients, we constructed a 16-dimensional Hadamard matrix and discarded the first 6 columns. Note that 2^4^ is the smallest power of 2 that is larger than *m*, and this specifies a 2^−6^-replicate of the full factorial, the latter consisting of all 2^10^ treatments. Let *H* denote the resulting 16 × 10 matrix. Each row of *H* specifies a combination of values to be assigned to the missing entries of variables *x*_·2_ and *x*_·3_, and variables *x*_·5_ to *x*_·12_. For instance, since the top row of *H* consists only of ones, missing entries of *x*_·*j*_ are replaced by $\max\{x_{ij}\;:\;i\in \overset{\circ }{I}\}$. The second row of *H* is an alternating sequence of −1 and 1, specifying that the missing entries of *x*_·2_ be replaced by $\min\left\{x_{i2}\;:\;i\in \overset{\circ }{I}\right\}$, those of *x*_·3_ be replaced by $\max\{x_{i3}\;:\;i\in \overset{\circ }{I}\}$, and so on.

For each of the 16 combinations of missingness, estimates of the logistic constant parameter and 18 regression coefficients were stored, alongside their estimated standard errors. Let *B* and *S* denote the resulting 16 × 19 matrices. In direct analogy with (2.1), the main effect of missingness of the *k*th partially observed variable on coefficient ℓ is constructed orthogonally to the other effects as $(h_{k}^{T}b_{\ell })/8$, where *h*_*k*_ is the *k*th column of *H* and *b*_ℓ_ is the ℓth column of *B*. Let *E*_*B*_ denote the 10 × 19 matrix of such effects. The same analysis using *S* in place of *B* produces a matrix *E*_*S*_ of main effects of missingness on the standard error estimates. The entries of *E*_*B*_ and *E*_*S*_ are best calibrated against the entries of *B* and *S*, respectively. For instance, dividing the absolute entries in the ℓth column of *E*_*B*_ by $\max_{c}|{\hat{\beta }}_{\ell }^{\left(c\right)}|$ produces a dimension-free number, comparable across rows and columns and between tables for coefficients and standard errors. These are presented in tables 2 and 3. We do not put forward any definitive thresholds. As in other contexts, the appropriate exposition presents the evidence in an incisive form, avoiding binary decisions to the extent feasible.

**Table 2.** Absolute main effects of missingness in variable *x*_·*j*_ on the estimated logistic regression coefficients ${\hat{\beta }}_{k}$ relative to the maximum absolute coefficient estimate for each column.

	${\hat{\beta }}_{0}$	${\hat{\beta }}_{1}$	${\hat{\beta }}_{2}$	${\hat{\beta }}_{3}$	${\hat{\beta }}_{4}$	${\hat{\beta }}_{5}$	${\hat{\beta }}_{6}$	${\hat{\beta }}_{7}$	${\hat{\beta }}_{8}$	${\hat{\beta }}_{9}$	${\hat{\beta }}_{10}$	${\hat{\beta }}_{11}$	${\hat{\beta }}_{12}$	${\hat{\beta }}_{13}$	${\hat{\beta }}_{14}$	${\hat{\beta }}_{15}$	${\hat{\beta }}_{16}$	${\hat{\beta }}_{17}$	${\hat{\beta }}_{18}$
*x*_·2_	0.1089	0.5169	0.3948	0.1455	0.0593	0.0502	0.1086	0.0285	0.0693	0.0473	0.0524	0.0697	0.0771	0.1277	0.0936	0.0845	0.0757	0.2560	0.0498
*x*_·3_	0.0677	0.5609	0.2976	0.5676	0.4488	0.1012	0.0002	0.1194	0.4706	0.5574	0.3038	0.2773	0.0729	0.0269	0.0155	0.1321	0.2632	0.1075	0.4811
*x*_·5_	0.0487	0.0367	0.0599	0.0949	0.1875	0.3416	0.2281	0.0995	0.0517	0.0491	0.0382	0.0628	0.0294	0.0752	0.0289	0.3551	0.0157	0.0817	0.0361
*x*_·6_	0.0353	0.0514	0.1006	0.0034	0.1829	0.3288	0.5565	0.0394	0.0031	0.0069	0.0136	0.1156	0.1167	0.0875	0.0090	0.0059	0.0227	0.2064	0.0615
*x*_·7_	0.0134	0.0143	0.0087	0.0004	0.0367	0.0127	0.0023	0.0779	0.0148	0.0195	0.0326	0.0174	0.0019	0.0435	0.0135	0.0767	0.0121	0.0015	0.0167
*x*_·8_	0.0307	0.1179	0.0088	0.0254	0.0436	0.0022	0.0178	0.0389	0.9496	0.8474	0.5734	0.1171	0.0694	0.1603	0.0693	0.3356	0.1270	0.1300	0.0339
*x*_·9_	0.1765	0.0987	0.0129	0.0220	0.1336	0.0440	0.0801	0.1110	0.5834	0.7935	0.3536	0.0493	0.0227	0.0906	0.0915	0.4983	0.0806	0.0382	0.1506
*x*_·10_	0.0196	0.0342	0.0134	0.0010	0.1635	0.0130	0.0277	0.0157	0.0651	0.0519	0.0590	0.0230	0.0240	0.1004	0.0089	0.1985	0.0477	0.0441	0.0056
*x*_·11_	0.1275	0.0249	0.0207	0.0585	0.0097	0.0574	0.0402	0.0150	0.0041	0.0003	0.0092	0.5274	0.0390	0.0014	0.0317	0.2230	0.0860	0.0904	0.0121
*x*_·12_	0.0513	0.1529	0.0970	0.0314	0.1482	0.1870	0.1053	0.2996	0.0754	0.0588	0.0335	0.2392	0.3229	0.0589	0.0252	0.1013	0.1039	0.5435	0.0227

**Table 3.** Absolute main effects of missingness in variable *x*_·*j*_ on the standard errors of ${\hat{\beta }}_{k}$ relative to the maximum standard error for each column.

	${\hat{\beta }}_{0}$	${\hat{\beta }}_{1}$	${\hat{\beta }}_{2}$	${\hat{\beta }}_{3}$	${\hat{\beta }}_{4}$	${\hat{\beta }}_{5}$	${\hat{\beta }}_{6}$	${\hat{\beta }}_{7}$	${\hat{\beta }}_{8}$	${\hat{\beta }}_{9}$	${\hat{\beta }}_{10}$	${\hat{\beta }}_{11}$	${\hat{\beta }}_{12}$	${\hat{\beta }}_{13}$	${\hat{\beta }}_{14}$	${\hat{\beta }}_{15}$	${\hat{\beta }}_{16}$	${\hat{\beta }}_{17}$	${\hat{\beta }}_{18}$
*x*_·2_	0.0086	0.0249	0.3772	0.0701	0.0029	0.0001	0.0092	0.0007	0.0668	0.0570	0.0708	0.0067	0.0687	0.0015	0.0082	0.0060	0.0034	0.0595	0.0099
*x*_·3_	0.0003	0.0129	0.0515	0.2387	0.0191	0.0083	0.0115	0.0177	0.4602	0.4557	0.2739	0.0094	0.0121	0.0114	0.0085	0.0075	0.0124	0.0169	0.0141
*x*_·5_	0.0006	0.0069	0.0013	0.0126	0.0007	0.0043	0.0123	0.0040	0.0077	0.0076	0.0043	0.0014	0.0294	0.0062	0.0019	0.0048	0.0016	0.0119	0.0111
*x*_·6_	0.0019	0.0066	0.0112	0.0114	0.0071	0.0022	0.0335	0.0008	0.0003	0.0005	0.0020	0.0001	0.0022	0.0035	0.0005	0.0011	0.0004	0.0086	0.0076
*x*_·7_	0.0003	0.0021	0.0003	0.0016	0.0023	0.0016	0.0008	0.0006	0.0222	0.0195	0.0218	0.0003	0.0022	<10^−4^	0.0002	0.0012	<10^−4^	0.0022	0.0016
*x*_·8_	0.0034	0.0044	0.0008	0.0066	0.0111	0.0012	0.0039	0.0057	0.0329	0.0419	0.0120	0.0049	0.0051	0.0098	0.0139	0.0024	0.0054	0.0048	0.0010
*x*_·9_	0.0101	0.0012	0.0023	0.0027	0.0122	0.0019	0.0006	0.0024	0.0493	0.0725	0.0333	0.0000	0.0051	0.0100	0.0019	0.0002	0.0008	0.0045	0.0055
*x*_·10_	0.0043	0.0002	0.0019	0.0032	0.0013	0.0019	0.0024	0.0032	0.0225	0.0218	0.0275	0.0009	0.0060	0.0016	0.0002	0.0039	0.0006	0.0054	0.0005
*x*_·11_	0.0092	0.0008	0.0074	0.0025	0.0017	0.0008	0.0003	0.0032	0.0006	0.0007	0.0019	0.0218	0.0129	0.0010	0.0023	0.0025	0.0033	0.0006	0.0021
*x*_·12_	0.0057	0.0092	0.0120	0.0031	0.0150	0.0123	0.0032	0.0196	0.0063	0.0063	0.0042	0.0049	0.0037	0.0020	0.0067	0.0030	0.0017	0.0169	0.0032

Missingness in variables *x*_·3_ and *x*_·8_ has an appreciable effect on several of the coefficient estimates. Some of the standard errors are also affected, particularly those on ${\hat{\beta }}_{j}$, *j* ∈ {8, 9, 10}. Other standard errors are relatively unaffected by the extreme reassignments of missing entries, typically varying by less than 5% and in many cases less than 1%. For the more stable coefficient estimates, namely, ${\hat{\beta }}_{0}$, ${\hat{\beta }}_{7}$, ${\hat{\beta }}_{13}$, ${\hat{\beta }}_{14}$ and ${\hat{\beta }}_{16}$, and their standard errors, it is reasonable to interpret the output from an arbitrary treatment assignment from the factorial combination. Such values are reported in table 4.

**Table 4.** Estimates and estimated standard errors of logistic regression coefficients (additive on the logit scale).

*j*	${\hat{\beta }}_{j}$	standard error	*p*-value
0	−1.45	1.07	0.176
7	0.384	0.36	0.293
13	0.296	0.57	0.602
14	0.425	0.40	0.293
16	0.154	0.34	0.646

None of the variables studied is statistically significant at typical thresholds, the strongest suggestion coming from *x*_·7_ and *x*_·14_. It is also worth noting that none of the omitted rows from table 4 would be deemed statistically significant according to their notional *p*-values, which are all in excess of 0.39.

The analysis was repeated using a different 2^−6^ fraction obtained by multiplying the Hadamard matrix, or equivalently the reduced form *H*, by minus one. The most notable differences were on the relative effect of missingness of variable *x*_·3_ on ${\hat{\beta }}_{2}$ and ${\hat{\beta }}_{7}$, which were 0.374 and 0.301, respectively, in the second replicate, compared with 0.298 and 0.119 in the first replicate.

### 3.2. A sociological example

The data to be analyzed, from the US National Longitudinal Study of Youth (1979), were used by Battey *et al.* [8] to illustrate different statistical issues to those in the present article. The binary outcome, coded as {0, 1}, is enrolment on a 4-year-degree-granting institution for at least 1 year. Five explanatory variables are as follows: ability, measured as the respondent’s score on the Armed Forces Qualifying Test, administered to all respondents in the 1981 wave of the survey; family income in childhood, measured as the log of total net family income in 1979; sex, as indicated by the respondent; race, recorded by interviewer observation; and whether respondents were living with at least one parent at the time of the first survey. The sample was restricted to those respondents who were classified as black or non-black and non-Hispanic. These data are summarized in table 5.

**Table 5.** Summary of data.

covariate	description	sample range	% missing
*x*_·1_	sex	{1 = male, 0 = female}	0
*x*_·2_	AFQT score	percentage (0−100)	4.3
*x*_·3_	log income	continuous (3.00−11.23)	51.2
*x*_·4_	race	{1 = black, 0 = non-black/non-Hispanic}	0
*x*_·5_	lives with parent	{1 = yes, 0 = no}	5.1

With only three variables having missing observations, it is feasible to report the output from all 2^3^ factorial combinations. However, to illustrate the previous ideas, we calculate the main effects of missingness from the last three columns of the eight-dimensional standard Hadamard matrix. Information analogous to that in tables 2 and 3 is given in tables 6 and 7. The coefficient estimate ${\hat{\beta }}_{3}$ is highly unstable and to a lesser extent so is ${\hat{\beta }}_{5}$. Other coefficients are more secure and, for these, we report the output from an arbitrary treatment combination in table 8.

**Table 6.** Main effects of missingness in variable *x*_·*j*_ on the estimated logistic regression coefficients ${\hat{\beta }}_{k}$ relative to the maximum absolute coefficient estimate for each column.

	${\hat{\beta }}_{0}$	${\hat{\beta }}_{1}$	${\hat{\beta }}_{2}$	${\hat{\beta }}_{3}$	${\hat{\beta }}_{4}$	${\hat{\beta }}_{5}$
*x*_·2_	0.042	0.053	0.154	0.048	0.150	0.322
*x*_·3_	0.293	0.012	0.0060	1.008	0.021	0.051
*x*_·5_	0.024	0.018	0.0011	0.031	0.0074	0.468

**Table 7.** Main effects of missingness in variable *x*_·*j*_ on the estimated standard error of ${\hat{\beta }}_{k}$ relative to the maximum standard error for each column.

	${\hat{\beta }}_{0}$	${\hat{\beta }}_{1}$	${\hat{\beta }}_{2}$	${\hat{\beta }}_{3}$	${\hat{\beta }}_{4}$	${\hat{\beta }}_{5}$
*x*_·2_	0.021	0.032	0.097	0.019	0.031	0.033
*x*_·3_	0.625	0.0015	0.0014	0.625	0.0060	0.017
*x*_·5_	0.0042	0.0007	0.0007	0.0073	0.0024	0.064

**Table 8.** Estimates and estimated standard errors of logistic regression coefficients (additive on the logit scale).

*j*	${\hat{\beta }}_{j}$	standard error	*p*-value
0	−3.39	0.250	<10^−9^
1	−0.366	0.049	<10^−9^
2	0.0422	0.0010	<10^−9^
4	1.051	0.064	<10^−9^

## 4. Discussion

When observations on explanatory variables are missing, a reasonable approach for general use is to assess the sensitivity of the inference to this missingness. Fragility would point to limitations of statistical inference on the data at hand. This is in contrast with the widely deployed strategy of multiple imputation [13] in which missing entries are replaced by values drawn multiple times from a distribution, and the estimates and standard errors averaged. This produces a single answer without warning.

The approach here is semi-descriptive: an acknowledgement that statistics can only take us so far when the quality of the data is low. Any more formal statistical guarantees would entail strong assumptions on the process by which the missingness arises. While conceptually very different, it is possible that aspects of the proposal are operationally related to tests of the so-called Missing Completely at Random (MCAR) assumption.

## Figures and Tables

**Figure 1.**
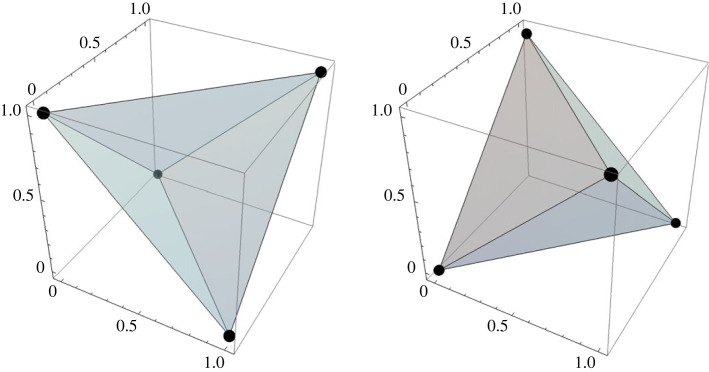
Convex hull of the four treatment combinations specified by the two half-replicates of the 2^3^ factorial.

## Data Availability

The bone marrow data and source code for reproducing the analysis are available from Dryad: https://doi.org/10.5061/dryad.vq83bk3vp [14].

## Appendix A. Some geometric properties of least squares in the full factorial system

The recommended sensitivity analysis is conditional on the data, and it is not in keeping with the article to introduce any mechanism through which the factorial contrasts could be treated as random. It is, however, of some interest to know how the set of coefficients arising from the factorial or fractional factorial treatment plan relates to those that would have been obtained had the data been available in their entirety.

When the number of covariates subject to missing observations is relatively small (less than four, say), it is feasible and reasonable to report the output from a full factorial arrangement. The present section provides a geometric characterization of the relationship between the notional regression coefficient estimate, $\hat{\beta }$ say, and those observable ones determined by substitution at each factorial combination of missingness. The discussion is restricted to linear least-squares estimates so that $\hat{\beta }={\left(\sum x_{i}x_{i}^{T}\right)}^{-1}\sum x_{i}y_{i}$ and ${\hat{\beta }}^{\left(1\right)},\ldots ,{\hat{\beta }}^{\left(2^{m}\right)}$ are analogously defined.

While one might expect the notional coefficient estimate $\hat{\beta }$ to reside in the convex hull of ${\hat{\beta }}^{\left(1\right)},\ldots ,{\hat{\beta }}^{\left(2^{m}\right)}$, this strong property has been neither proved nor refuted in the current work (see the discussion below proposition A.1). Instead it has been demonstrated that $\hat{\beta }$ is in the convex hull of a closely related set, which cannot be explicitly calculated from the observed data.

While worth reporting, the connection to §2 is slight because the geometric result is much stronger than is required to justify the sensitivity analysis.

The proof of proposition A.1 is in appendix B.

Proposition A.1. *Provided that* $w=\sum_{i}x_{i}x_{i}^{T}$ *is invertible, and the missing entries in each column of* $\overset{\circ }{X}$ *are not one of the two extreme points from the corresponding column of* *X*, *the notional least-squares estimate satisfies*

A 1 $$\hat{\beta }\in \mathrm{conv}\{w^{-1}w^{\left(c\right)}{\hat{\beta }}^{\left(c\right)}\;:\;c=1,\ldots ,2^{m}\}\subset R^{d},$$

*where* $w^{\left(c\right)}=\sum_{i}{\overset{\circ }{x}}_{i}^{\left(c\right)}{\overset{\circ }{x}}_{i}^{(c)T}$ *and, for a finite set* A, conv(A) *is the convex hull of* A.

Note that ${\overset{\circ }{X}}^{\left(c\right)}=X+P^{\left(c\right)}$, say, where *P*^(*c*)^ is a matrix consisting primarily of zeros except in the positions corresponding to missing entries of *X*, where they take values $\;p_{ij}^{\left(c\right)}=\max\{x_{kj}\;:\;k\in \overset{\circ }{I}\}-x_{ij}$ or $\;p_{ij}^{\left(c\right)}=\min\{x_{kj}\;:\;k\in \overset{\circ }{I}\}-x_{ij}$ depending on the assignment prescribed by *c*. Thus,

$$w^{-1}w^{\left(c\right)}=I_{n}+{\left(X^{T}X\right)}^{-1}\left[P^{(c)T}X+X^{T}P^{\left(c\right)}+P^{(c)T}P^{\left(c\right)}\right]=I_{n}+A,$$

say, where *I*_*n*_ is the *n*-dimensional identity matrix. It follows that *w*^−1^*w*^(*c*)^ − *I*_*n*_ is positive semi-definite if *A* is. Whether this is satisfied is not immediately clear, as some configurations produce a constituent matrix *P*^(*c*)T^ *X* + *X*^T^*P*^(*c*)^ that is negative definite.

Since §2 suggests fractional factorial assessments of missingness, it is of some interest to consider whether a version of (A1) holds with *c* varying over a restricted set. Again, the semi-descriptive assurances of §2 do not hinge on geometric results of this nature, which are much stronger.

Inspection of the proof of proposition A.1 reveals that equation (A1) holds for any subset $K\subset \{1,\ldots ,2^{m}\}$ of the full factorial set such that $x_{i}\in \mathrm{conv}\{{\overset{\circ }{x}}_{i}^{\left(c\right)}\;:\;c\in K\}$ for all *i*. Thus, if one considers K=F for some fractional factorial combination, the aforementioned is in effect a restriction on the values of the missing entries of each *x*_*i*_, since $\mathrm{conv}\{{\overset{\circ }{x}}_{i}^{\left(c\right)}\;:\;c\in F\}\subset \mathrm{conv}\{{\overset{\circ }{x}}_{i}^{\left(c\right)}\;:\;c=1,\ldots ,2^{m}\}$. By Caratheodory’s theorem (lemma B.1),

$$\text{conv}\{{\overset{\circ }{x}}_{i}^{\left(c\right)}\;:\;c=1,\ldots ,2^{m}\}=\text{conv}\{s_{i1},\ldots ,s_{i(d+1)}\}=\text{conv}(S_{i}),$$

where *s*_*ij*_ are vectors in the finite set $\left\{{\overset{\circ }{x}}_{i}^{\left(c\right)}\;:\;c=1,\ldots ,2^{m}\right\}$. Let $C_{i}$ denote the set of indices *c* ∈ {1, …, 2^*m*^} corresponding to $S_{i}$. Then

A 2 $$\hat{\beta }\in \text{conv}\{w^{-1}w^{\left(c\right)}{\hat{\beta }}^{\left(c\right)}\;:\;c\in K\}$$

would hold with $K=\cup_{i=1}^{n}C_{i}$.

To explore the strength of the condition $x_{i}\in \mathrm{conv}\{{\overset{\circ }{x}}_{i}^{\left(c\right)}\;:\;c\in K\}$ when K=F, a set defining a fractional factorial arrangement, consider *m* = *d* so that the index *i* can be dropped in $\mathrm{conv}\{{\overset{\circ }{x}}_{i}^{\left(c\right)}\;:\;c\in F\}$, and ${\overset{\circ }{x}}^{\left(c\right)}$ represents a vertex in the design space. For instance, with *m* = *d* = 3, $\max\{x_{ij}\;:\;i\in \overset{\circ }{I}\}=1$ and $\min\{x_{ij}\;:\;i\in \overset{\circ }{I}\}=0$ for *j* = 1, 2, 3, the vertices corresponding to F and $\bar{F}$ are {(1, 1, 1), (1, 0, 0), (0, 1, 0), (0, 0, 1)} and {(0, 0, 0), (1, 0, 1), (0, 1, 1), (1, 1, 0)}, respectively. Figure 1 depicts $\mathrm{conv}\{{\overset{\circ }{x}}^{\left(c\right)}\;:\;c\in F\}$ and $\mathrm{conv}\{{\overset{\circ }{x}}^{\left(c\right)}\;:\;c\in \bar{F}\}$ for these two half-replicates of the 2^3^ factorial.

It is visually clear that the intersection B, say, of $\mathrm{conv}\{{\overset{\circ }{x}}^{\left(c\right)}\;:\;c\in F\}$ and $\mathrm{conv}\{{\overset{\circ }{x}}^{\left(c\right)}\;:\;c\in \bar{F}\}$ is rather small, so that $x_{i}\in B$ for all *i* is a strong requirement, especially for large *m*. The conclusion is only amplified when higher levels of fractionation are used. It follows that equation (A 2) will rarely be satisfied simultaneously when K is taken as any of the relevant fractional factorial sets.

While worth pointing out, this conclusion is immaterial for assessing the sensitivity to missingness as detailed in §2.

## Appendix B. Proofs

**B.1. Preliminary lemmata**

Proofs of the two important results in convex analysis, lemmas B.1 and B.2, can be found in the stated references. These lemmas will be used in the proof of proposition A.1, together with lemma B.3, proved here.

Lemma B.1 Caratheodory’s convex hull theorem (e.g. [15], theorem 17.1). *Let* A *be any set of points and directions in* $R^{d}$, *and let* C=conv(A). *Then* z∈C *if and only if* *z* *can be expressed as a convex combination of* *d* + 1 *of the points and directions in* A (*not necessarily distinct*).

Lemma B.2 Krein and Šmulian ([16], theorem 3). *For sets* A *and* B, *denote their Minskowski sum by* A+B={a+b∣a∈A,b∈B}. *Minskowski summation commutes with the formation of convex hulls, i.e*.: conv(A+B)=conv(A)+conv(B).

Lemma B.3. *Let* A *and* B *be finite subsets of* $R^{d}$ *and* A×B *their Cartesian product. Then* conv(A×B)=conv(A)×conv(B).

Proof. One inclusion, conv(A×B)⊆conv(A)×conv(B), is evident. To prove the converse, let (a,b)∈conv(A)×conv(B). By lemma B.1, there exists $a_{1},\ldots ,a_{d+1}\in A$ and $b_{1},\ldots ,b_{d+1}\in B$ such that $a=\sum_{\;j}\lambda_{\;j}a_{j}$ and $b=\sum_{\;j}\gamma_{j}b_{j}$, where *λ*_*j*_, *γ*_*j*_ ≥ 0 for all *j* and $\sum_{\;j}\lambda_{\;j}=\sum_{\;j}\gamma_{j}=1$. Write

$$\begin{array}{rl} \left(a,b\right) & =\left(\sum_{\;j=1}^{d+1}\lambda_{j}a_{j},\sum_{\;j=1}^{d+1}\gamma_{j}b_{j}\right)=\left\{\sum_{k=1}^{d+1}\gamma_{k}\left(\sum_{\;j=1}^{d+1}\lambda_{j}a_{j}\right),\sum_{k=1}^{d+1}\lambda_{j}\left(\sum_{\;j=1}^{d+1}\gamma_{j}b_{j}\right)\right\}\\ & =\sum_{\;j,k}\lambda_{j}\gamma_{k}(a_{j},b_{k}). \end{array}$$

Since $\sum_{\;j,k}\lambda_{j}\gamma_{k}=1$, (a,b)∈conv(A×B). Thus, conv(A)×conv(B)⊆conv(A×B). ▪

**B.2. Proof of proposition A.1**

Proof. Let $X_{i}=\{{\overset{\circ }{x}}_{i}^{\left(c\right)}\;:\;c=1,\ldots ,2^{m}\}$ and $M_{i}=\{{\overset{\circ }{x}}_{i}^{\left(c\right)}{\overset{\circ }{x}}_{i}^{(c)T}\;:\;c=1,\ldots ,2^{m}\}$. By the constraint on the missing entries of $\overset{\circ }{X}$ relative to the two extremes of the corresponding column of *X*, $x_{i}\in \mathrm{conv}(X_{i})$ and $\left(x_{i},x_{i})\in \mathrm{conv}(X_{i}\times X_{i})=\mathrm{conv}(X_{i})\times \mathrm{conv}(X_{i}\right)$ by lemma B.3. Thus, since the tensor product is a surjective bilinear map from $R^{d}\times R^{d}$ to $R^{d}⊗R^{d}$, $x_{i}x_{i}^{T}\in \mathrm{conv}(M_{i})$. By the definition of the Minkowski sum, $\sum_{i}x_{i}x_{i}^{T}\in \sum_{i}\mathrm{conv}(M_{i}),$ which is equal to $\mathrm{conv}(\sum_{i}M_{i})$ by lemma B.2. Let $S_{i}=\{{\overset{\circ }{x}}_{i}^{\left(c\right)}y_{i}\;:\;c=1,\ldots ,2^{m}\}$. By lemma B.1, there exists vectors $s_{i1},\ldots ,s_{i(d+1)}\in X_{i}$ such that $x_{i}=\sum_{\;j}\lambda_{ij}s_{ij}$, where $\sum_{\;j}\lambda_{ij}=1$ and *λ*_*ij*_ ≥ 0 for all *i* ∈ {1, …, *n*} and all *j* ∈ {1, …, *d* + 1}. It follows immediately that $x_{i}y_{i}\in \mathrm{conv}(S_{i})$, and from lemma B.2 that $\sum_{i}x_{i}y_{i}\in \mathrm{conv}(\sum_{i}S_{i})$. Let $w=\sum_{i}x_{i}x_{i}^{T}$ and $w^{\left(c\right)}=\sum_{i}{\overset{\circ }{x}}_{i}^{\left(c\right)}{\overset{\circ }{x}}_{i}^{(c)T}$. By the definition of the least-squares estimator, $w\hat{\beta }=\sum_{i=1}^{n}x_{i}y_{i}$, the right-hand side of which belongs to $\mathrm{conv}(\sum_{i}S_{i})$. Since for all *c* ∈ {1, …, 2^*m*^}, $w^{\left(c\right)}{\hat{\beta }}^{\left(c\right)}=\sum_{i=1}^{n}{\overset{\circ }{x}}_{i}^{\left(c\right)}y_{i}$, it follows that

B 1 $$w\hat{\beta }\in \text{conv}(Q),$$

where

$$Q=\{w^{\left(c\right)}{\hat{\beta }}^{\left(c\right)}\;:\;c=1,\ldots ,2^{m}\}.$$

For any q∈conv(Q), there exists $q_{1},\ldots ,q_{\left(d+1\right)}\in Q$ such that $q=\sum_{j}\alpha_{j}q_{j}$ by lemma B.1, where *α*_*j*_ ≥ 0 for all *j* ∈ {1, …, *d* + 1} and $\sum_{j}\alpha =1$. Thus, $w^{-1}q=\sum_{j}\alpha_{j}w^{-1}q_{j}$, showing that any *w*^−1^*q* for q∈Q belongs to

$$\text{conv}\{w^{-1}w^{\left(c\right)}{\hat{\beta }}^{\left(c\right)}\;:\;c=1,\ldots ,2^{m}\}.$$

The conclusion follows by setting $q=w\hat{\beta }$. ▪
